# Three dimensional right ventricular diastolic vortex rings: characterization and comparison with left ventricular diastolic vortex rings from 4D flow MRI

**DOI:** 10.1186/1532-429X-16-S1-P42

**Published:** 2014-01-16

**Authors:** Mohammed S ElBaz, Emmeline Calkoen, Jos J Westenberg, Boudewijn PF Lelieveldt, Arno Roest, Rob J van der Geest

**Affiliations:** 1 Division of Image Processing, Radiology, Leiden University Medical Center (LUMC), Leiden, Netherlands; 2 Intelligent Systems, Delft University of Technology, Delft, Netherlands; 3 Pediatric Cardiology, Leiden University Medical Center, Leiden, Netherlands

## Background

Efficient right ventricular (RV) pumping function requires optimal blood flow dynamics. In the left ventricle (LV), diastolic vortex ring formation distal to the mitral valve (MV) has been reported to be an important mechanism for such blood flow optimization. Earlier work based on computational fluid dynamics (CFD) simulations using simplified RV geometry modeling have reported vortex ring formation in the RV during the early filling phase and its breakdown at the late diastolic phase. However, neither those CFD studies have characterized vortex rings nor have they been confirmed by 4D flow MRI. The purpose of this study was to investigate and characterize the formation of vortex rings during diastolic filling in the RV and to compare them with those of LV in healthy volunteers.

## Methods

Ten healthy volunteers (age: 20 ± 7 years) underwent three-dimensional (3D), time resolved, three-directional velocity-encoded MRI at 3T (Philips). MRI was performed in a 3D isotropic dataset of 4.2 × 4.2 × 4.2 mm 3 with whole heart coverage. Retrospective gating with 30 phases reconstructed and velocity sensitivity of 150 cm/s in all directions was used. The Lambda2 (λ2) method was used to extract the 3D vortex structures inside the RV at the phases of early (E) and late (A) filling. The most circular and compact ring was extracted from each phase. The location of a vortex ring was characterized by its longitudinal position (L) and its orientation (Table 1). The circularity of the vortex ring shape was quantified using a circularity index (CI). RV vortex ring parameters were compared to those of LV vortex ring. For the LV vortex rings, all mentioned parameters were measured relative to the MV and LV geometry.

**Table 1:** Characterization of both early and late RV diastolic vortex rings in comparison to LV diastolic vortex rings

Chamber		RV	LV	Statistical Significance
**parameter**	**phase**			

**L^(1) ^**	**E**	0.90 ± 0.05	0.83 ± 0.04	p < 0.05

	**A**	0.88 ± 0.04	0.85 ± 0.04	p < 0.05

**Orientation** **(in degrees)^(2) ^**	**E**	64.46^ο ^± 5.78^ο^	69.23^ο ^± 7.97^ο^	NS

	**A**	67.36^ο ^± 7.71^ο^	71.53^ο ^± 4.30^ο^	NS

**CI^(3) ^**	**E**	0.67 ± 0.10	0.80 ± 0.07	p < 0.05

	**A**	0.54 ± 0.12^(s)^	0.71 ± 0.08^(s)^	p < 0.05

**(1) L **: the normalized distance of the vortex ring center from the RV/LV apex (normalized by the RV long axis length which defined as the line from the mid of the annulus (i.e.TV or MV) to the apex of the ventricle) **(2) Orientation**: the angle between the vortex's fitting plane and the RV/LV long axis **(3) CI**: the ratio between the short axis to the long axis diameter of the ring **(s)**: The parameter was significantly different (p < 0.05) in ventricle's A-filling vortex rings compared to E-filling vortex rings.

## Results

**Figure 1:**
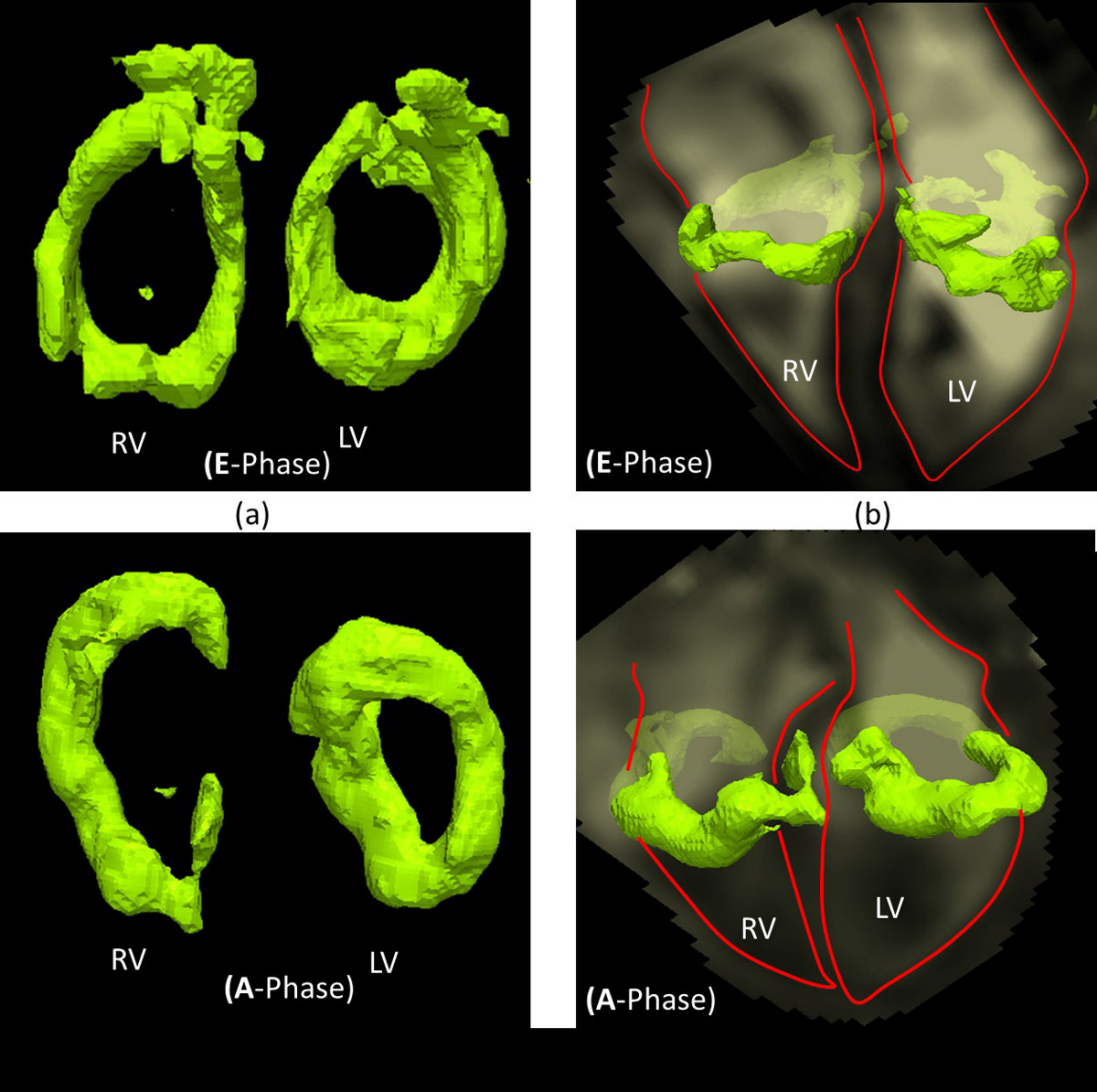
**A: Lambda2 based extracted isosurface of RV and LV quasi-donut shaped vortex rings identified during the early filling phase**. B: Corresponding E-phase vortex positions relative to the cardiac geometry in a long axis view (ventricular and atrial boundaries are outlined in red). C: RV arch-shaped rings and LV elongated rings identified during the late filling phase. D: Corresponding A-phase vortex positions relative to the cardiac geometry in a long axis view.

In all subjects, the formation of a compact vortex ring was observed distal to the tricuspid annulus during the E-phase and another vortex ring was formed during the A-phase. The E-filling vortex ring tended to have a more compact and quasi-donut shape compared to an incomplete, arch-shaped A-filling ring (Figure 1). The locations and orientations of both E- and A- rings were not significantly different. However, the E- ring shape was significantly more circular than the (extrapolated) A-filling ring. Compared to the LV vortex rings, the RV rings' relative locations were significantly closer to the annulus but with similar orientation with respect to the ventricle's long axis. Both E-and A-filling RV rings were significantly more elliptical than their corresponding LV rings (Table 1).

## Conclusions

As opposed to previous results from CFD simulations, our 3D vortex analysis from 4D FLOW MRI revealed the formation of RV diastolic vortex rings during both E and A filling phases and not only during the E phase. RV vortex rings have similar orientation as LV vortex rings but demonstrated significantly different relative position and shape circularity. Our results suggest that RV diastolic vortex rings might play a similar role in diastolic blood flow optimization as that of the LV rings.

## Funding

Dutch Technology Foundation (STW): project number 11626.

